# Recent Developments in New Therapeutic Agents against Alzheimer and Parkinson Diseases: In-Silico Approaches

**DOI:** 10.3390/molecules26082193

**Published:** 2021-04-11

**Authors:** Pedro Cruz-Vicente, Luís A. Passarinha, Samuel Silvestre, Eugenia Gallardo

**Affiliations:** 1 CICS-UBI, Health Sciences Research Centre, University of Beira Interior, 6201-001 Covilhã, Portugal; pedromvcruz@hotmail.com; 2 UCIBIO—Applied Molecular Biosciences Unit, Department of Chemistry, Faculty of Sciences and Technology, NOVA University Lisbon, 2829-516 Caparica, Portugal; 3 Laboratory of Pharmaco-Toxicology—UBIMedical, University of Beira Interior, 6200-001 Covilhã, Portugal; 4 CNC—Center for Neuroscience and Cell Biology, University of Coimbra, 3004-504 Coimbra, Portugal

**Keywords:** neurodegenerative diseases, Parkinson’s Disease, Alzheimer’s Disease, computer-aided drug design, in silico studies

## Abstract

Neurodegenerative diseases (ND), including Alzheimer’s (AD) and Parkinson’s Disease (PD), are becoming increasingly more common and are recognized as a social problem in modern societies. These disorders are characterized by a progressive neurodegeneration and are considered one of the main causes of disability and mortality worldwide. Currently, there is no existing cure for AD nor PD and the clinically used drugs aim only at symptomatic relief, and are not capable of stopping neurodegeneration. Over the last years, several drug candidates reached clinical trials phases, but they were suspended, mainly because of the unsatisfactory pharmacological benefits. Recently, the number of compounds developed using in silico approaches has been increasing at a promising rate, mainly evaluating the affinity for several macromolecular targets and applying filters to exclude compounds with potentially unfavorable pharmacokinetics. Thus, in this review, an overview of the current therapeutics in use for these two ND, the main targets in drug development, and the primary studies published in the last five years that used in silico approaches to design novel drug candidates for AD and PD treatment will be presented. In addition, future perspectives for the treatment of these ND will also be briefly discussed.

## 1. Introduction

The advances in medicine and the better quality of life of the general population have increased the average lifespan worldwide. According to a 2019 survey of the United Nations, 9% of the world’s population, the equivalent to 700 million people, is at/or above 65 years old and this number is expected to grow to at least 2 billion by 2050 [1]. Consequently, age-related diseases, where neurodegenerative conditions (ND) are included, are becoming more common and being recognized as a social problem in modern societies [2]. ND are characterized by a heterogeneous and progressive degeneration of the central and/or the peripheric nervous systems, as a consequence of the death of neuronal cells [3]. So far, hundreds of ND have been identified; however, each of them displays differences in terms of pathological characteristics, symptoms, and treatments [3]. Specifically, Alzheimer’s (AD) and Parkinson’s Disease (PD) are considered the most prevalent ND, affecting 11 and 5 in 1000 individuals more than 65 years old, respectively [4]. In fact, in 2020, more than 44 million and 10 million patients were diagnosed with AD and PD, respectively [4]. Despite the intense research and investment behind these disorders, their aetiology is still unknown, but it is thought to be caused by a combination of factors, such as the individual’s lifestyle and genetics, but also environmental factors, like exposure to toxins and pollution [4].

Indeed, several medical conditions, for instance primary neurological and neuropsychiatric diseases, are thought to contribute to dementia, mostly in elderly people, the age group with the highest dementia incidence. This condition can be developed as a consequence of some degree of degeneration, namely in the progression of the AD/PD diseases, vascular dementia, Lewy body accumulation, and frontotemporal lobar degeneration, among others. Moreover, mild cognitive impairment and dementia occurring across the individual’s lifespan might be related to chemotherapy-related cognitive dysfunction, vitamin deficiencies (e.g., B1, B12), normal pressure hydrocephalus, intracranial masses (e.g., subdural hematomas, brain tumors), traumatic brain injury, and psychiatric illness (depression major and anxiety) [5].

Unfortunately, there is no existing cure for these ND and the currently clinically used drugs aim only at symptomatic relief and are not capable of stopping neurodegeneration [6,7]. The biggest challenges in AD/PD drug development are the number of biological pathways and proteins involved in the diseases’ pathogenesis, the complexity of the affected organs (mostly the brain), and their aggressiveness [6,7]. Thus, the necessity of developing novel drugs has forced the pharmaceutical industry to employ new methodologies for the design of new compounds. The progresses in the biomolecular and structural fields allowed the determination of numerous three-dimensional (3D) structures of proteins, through nuclear magnetic resonance, X-ray crystallography, cryo-electron microscopy, among other techniques, and these have provided essential information about atomic interactions between protein-ligand [8]. The combination of computational and mathematical algorithms with the protein structural data has become increasingly used in modern medicinal chemistry to support drug design, generally defined as computer-aided drug design (CADD) [9]. These approaches can be subdivided into three main categories: sequence-based drug design, structure-based drug design (SBDD), and ligand-based drug design (LBDD) [9]. In particular, sequence-based drug design uses the protein sequence information deposited on the Protein Data Bank (PDB, https://www.rcsb.org/, accessed on 10 April 2021) database to build 3D homologue models of the protein 3D structure that can be further refined with molecular dynamics simulations [9]. The SBDD strategy is probably the most used in in silico studies; it uses the structural information found in the protein 3D structure to predict macromolecular binding sites and the affinity of ligands towards certain targets [9]. Within this strategy, molecular docking studies are the most known and used, including structure-based virtual screening, also known as target-based virtual screening, which allows for the selection of promising compounds from extensive libraries. Frequently, molecular dynamics are also used to obtain a more profound understanding of the interactions between ligands and macromolecules, as well as predicting the stability of the obtained binding poses. In most cases, a combination of these techniques is used in SBDD protocols [9]. Another methodology that is also commonly employed to study the affinity of ligands in targets that do not have a 3D structure available in the PDB is LBDD [9]. Similar to SBDD, this approach also allows the collection of the most relevant compounds from big libraries, and includes QSAR modeling, pharmacophore studies, similarity searching, among others [9]. Additionally, this approach can be combined with homology modeling [9]. These techniques have been improved over the years and are being increasingly used in drug development by researchers from academic centers and R&D of pharmaceutical companies, mostly in preliminary studies to select compounds with higher potential of success in further studies. Moreover, it is also possible to use software to filter molecules with positive ADMET (Absorption, Distribution, Metabolism, Excretion, and Toxicity) properties in addition to a predicted high affinity to the protein target [9]. Therefore, in this review, we intend to summarize the current therapeutics in use for these two ND conditions, the main targets being explored to develop new drugs in this context and, principally, the latest studies published in the last five years that employed in silico approaches to design potential drug candidates against AD and PD. Consequently, with this work, we aim to shed some light on relevant future perspectives in this challenging field for the treatment of these two very relevant ND.

## 2. Alzheimer’s Disease

AD is characterized by a slow and progressive decline in the cognitive functions and dementia, as a consequence of the loss of neurons, deterioration of the neurotransmission systems, and the accumulation of several proteins in the central nervous system (CNS) [10]. Overall, its pathophysiology features are the formation of amyloid plaques and neurofibrillary tangles in the brain, but more recently, dystrophic neurites, astrogliosis, neuropil threads, and microglial activation are also being reported [11]. Currently, only a few drugs are approved for clinical use in AD treatment and none of them can stop the progression of the disease, being mostly used in symptomatic therapy [12]. Despite all the scientific efforts made, more than 200 drug candidates have failed or been suspended from clinical trials in the last decade and no drug has been approved for AD treatment since 2003 [12]. These failures might be related to an inaccurate selection of the protein targets and the insufficient understanding of the complex etiology of AD [12]. In this topic, a characterization of the progresses accomplished in drug development employing computational approaches for the various protein targets involved in AD will be performed.

### 2.1. Acetylcholinesterase

**Figure 1:**
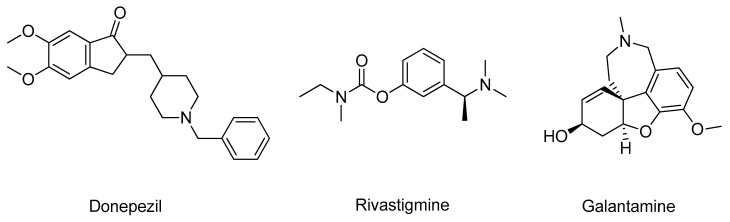
Structure of the clinically approved AChE inhibitors.

Acetylcholinesterase (AChE, E.C. 3.1.1.7) is an enzyme involved in the termination of impulse transmission by rapid hydrolysis of acetylcholine into choline and acetic acid [13]. In AD, the patient’s cholinergic systems endure extensive degeneration changes, leading to a hypofunction of the cholinergic neurons and a decline in the endogenous levels of acetylcholine [14]. Hence, AChE inhibitors are administered to counteract these effects in an attempt to decrease the breakdown rate of acetylcholine and restore its synaptic levels [15]. To date, only three AChE inhibitors are used in AD therapy, donepezil, rivastigmine, and galantamine (Figure 1); however, they only offer symptomatic relief and are mostly used to treat mild to moderate dementia [7]. Thus, new and more effective AChE inhibitors are needed [16].

**Figure 2:**
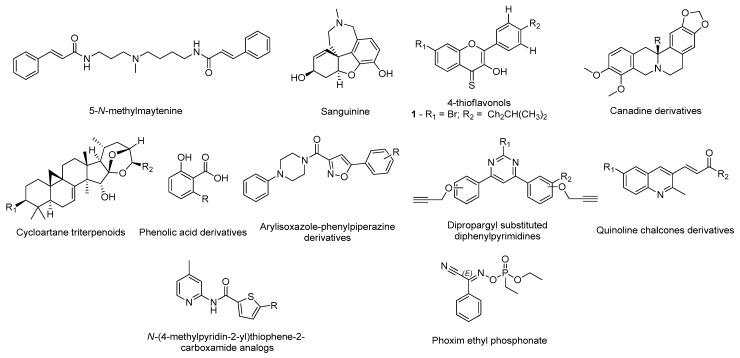
Structures of the studied AChE inhibitors.

Many researchers took advantage of the available 3D human AChE structures deposited in the PDB database and the information regarding the interactions with several ligands to carry out in silico methodologies to design potential inhibitors [17]. Structurally, the target has two distinct binding sites: one is peripheral and is situated at the entrance of the gorge and the other is located in the catalytic site [15]. Currently, researchers are focused in molecules that can occupy both binding sites and inhibit acetylcholine hydrolysis [17]. Grafov et al. studied the affinity of several naturally occurring alkaloids for both binding sites of AChE (PDB#6H12) using neostigmine, a known AChE inhibitor, as positive control [18]. The molecular docking results demonstrated that the alkaloid 5-*N*-methylmaytenine (Figure 2) could simultaneously bind to both binding sites, mainly by hydrophobic interactions, and therefore, could have a high pharmacological potential towards the design of novel AChE inhibitors [18]. A similar analysis was carried out by Ortiz and his research group for other alkaloid compounds extracted from plants of the *Hieronymiella* genus, but employing an additional step of molecular dynamic simulations to evaluate the binding modes to better understand in vitro results [19]. In this study, the compound sanguinine (Figure 2), structurally similar to galantamine (Figure 1), showed the most promising binding energy and interacted with residues Trp84, Gly117, Glu199, Ser200, Phe330, and His440. In addition, it presented high in vitro inhibitory potency for AChE (PDB#1DX6) as well, which can indicate that these amino acids are essential to promote higher inhibition rates [19]. Mughal and co-workers synthetized a series of 4-thioflavonols that displayed very promising in vitro outputs and studied their interactions with the AChE (PDB#4BDT) active site through molecular docking [20]. Particularly, compound 1 (Figure 2) had the highest affinity and also formed identical interactions with the amino acids of the catalytic site of AChE similar to the observed for donepezil, especially with residues Trp86 and Tyr337 [20]. An identical strategy was performed to study other natural products, namely canadine derivatives [21], cinnamic acid derivatives, indolinones and cycloartane triterpenoids [22], phenolic acid derivatives [23], and synthetic compounds, such as arylisoxazole-phenylpiperazine derivatives [24], dipropargyl substituted diphenylpyrimidines [25], quinoline chalcone derivatives [26], and *N*-(4-methylpyridin-2-yl)thiophene-2-carboxamide analogs [27], as displayed in (Figure 2). Ranjan’s research group [28] studied the affinity of several organophosphate derivatives against AChE (PDB#1B41) using docking-based virtual screening combined with molecular dynamics simulations. The compounds were selected based on the interaction with the main residues of the catalytic triad, Ser203, Glu334, and His447 [28]. The top ranked ligand was phoxim ethyl phosphonate (Figure 2), displaying the highest binding energy with these residues and it was advanced for further in vitro studies [28].

**Figure 3:**
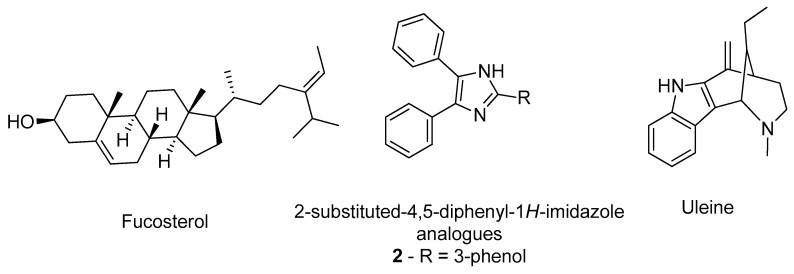
Structure of the studied AChE inhibitors.

Castro-Silva et al. studied the affinity of fucosterol (Figure 3) towards both binding sites of AChE (PDB#4EY7) and compared with the inhibitor neostigmine by docking and molecular dynamics to improve the analysis [29]. The results demonstrate that fucosterol has affinity towards both binding sites, by specifically interacting with the residues Trp286, Leu289, and Tyr341 of the peripheric site, and with the Trp86, Glu202, and Tyr449 of the AChE catalytic site, indicating that this compound might be a promising compound to advance for further studies [29]. Other researchers, namely Gurjar and co-workers, performed an in silico analysis in 2-substituted-4,5-diphenyl-1*H*-imidazole analogues (Figure 3) effecting a prediction of the compounds’ ADMET properties in addition to the molecular docking of the best ranked compounds, avoiding further testing of compounds with potential toxicity and unfavorable pharmacokinetics [30]. For instance, compound 2 (Figure 3) demonstrated the best results, being a potential candidate for further structural optimization for even better AChE inhibition [30]. Rocha and her group built and validated a machine learning model using pharmacophores based on the structures of more than 500 compounds with known and no inhibitory activity against AChE to predict the potential inhibitory activity of multiple indole alkaloids [31]. Of these, uleine (Figure 3) was predicted as being the alkaloid with the highest probability to present AChE inhibitory activity based on the in silico results [31]. The most promising compounds were further tested in vitro confirming the computational predictions regarding the AChE inhibition [31]. A distinct methodology was applied by Niu et al., building 2D- and 3D-QSAR models to classify molecules based on their potential to inhibit AChE, from a library of compounds that included known AChE inhibitors and non-inhibitors, with a predicted accuracy of 89.63% [32]. The most promising compounds were further tested by molecular docking to evaluate their affinity towards the target active site (PDB#1QTI) and the interaction with the residue Ser124 was demonstrated to be crucial for a higher affinity [32].

### 2.2. N-Methyl-D-aspartate Receptor

**Figure 4:**
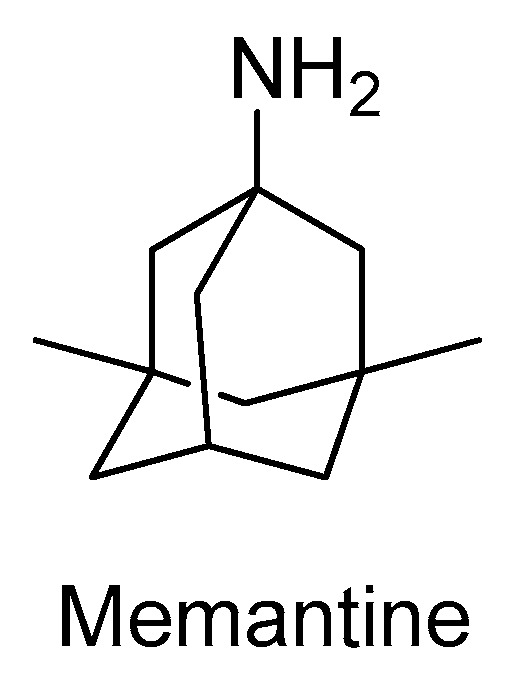
Structure of the clinically approved NMDAR antagonist.

The *N*-methyl-d-aspartate receptors (NMDAR) are a family of ligand-gated ionic membrane channels involved in non-selective cation transport and in the excitatory glutamatergic neurotransmission [33]. There are two types of NMDARs: the synaptic and the extra-synaptic receptors [33]. The synaptic are essential for synaptic plasticity and for the survival of neurons, while the extra-synaptic promote cell death and excitotoxicity, contributing for the etiology of AD [33]. The hyper-activation of the extra-synaptic NMDAR by glutamate, which is linked to an overproduction of free radicals and several enzymes that contribute to the deterioration of the CNS, can be controlled with NMDAR antagonists [34]. Currently, memantine (Figure 4) is the only NMDAR antagonist approved by the regulatory agencies for clinical use to treat moderate to severe dementia, selectively inhibiting the activity of extra-synaptic NMDARs [34]. In addition to its cognitive and functional pharmacological benefits, memantine has also demonstrated to slow down the emergence of further behavioral and psychotic manifestations. Furthermore, its administration resulted in significant increases in the extracellular concentrations of the neurotransmitters dopamine, norepinephrine, and their metabolites, demonstrating a biogenic amine neurotransmission enhancing effect useful in AD treatment [35].

**Figure 5:**
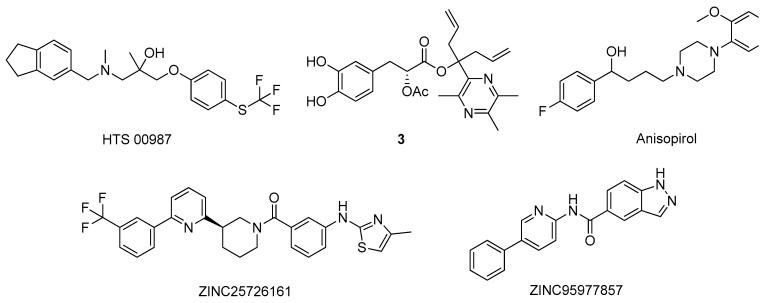
Structures of the studied NMDAR antagonists.

Nevertheless, over the years, numerous studies reported the design of novel active NMDAR antagonists using in silico methodologies [9]. For instance, Ivanova and co-workers used a virtual screening approach to discover new potential NMDAR antagonists [36]. Using a combination of various machine learning methods, including artificial neural networks and advanced multilinear techniques to build QSAR models, they screened over 13,000 natural compounds and ranked them based on their predicted affinity towards the target (PDB#5TP9) [36]. The best candidates were also analysed by docking and molecular dynamics simulations to identify essential structural moieties that could serve as basis for the design and development of novel and improved NMDAR antagonists [36]. A distinct strategy was carried out by Sharma et al. using pharmacophore modeling and a four-phased virtual screening study to identify potential drug candidates [37]. The pharmacophore model was generated and validated with a library of 40 known NMDAR antagonists, followed by screening, where the compounds were sorted based on the Lipinski’s rules and in terms of affinity towards the target [37]. Additionally, the hits were submitted to a docking analysis to fully validate the used methodology [37]. The main residues in the NMDAR active site are His88, Ser114, Thr116, Art121, Gly172, Ser173, Thr174, and Tyr214, of which the predicted compound HTS 00987 (Figure 5) interacts with His88, Thr174, and Tyr214, while memantine was not predicted to interact with none of these residues, indicating a potential pharmacological interest of this compound [37]. Waqar et al. built a homologue model of the 3D structure of NMADR based on the structure of the rat NMDAR (PDB#3JPW) and analyzed by molecular docking the affinity of several conantokins towards this target [38]. Moreover, most of the compounds interacted with residues Gln110 and Glu236, in the NR2B subunit of the NMDAR, and with Ile111, Phe114, and Pro177 by hydrophobic interactions [38]. A similar binding pattern was observed for the rat crystal structure, indicating that conankotins might be potential NMDAR antagonists [38]. In another study carried out by Hu and co-workers, the affinity of several tetramethylpyrazine derivatives, with known NMDAR antagonist activity, was studied towards its catalytic site (PDB#5UOW) by molecular docking [39]. Specifically, compound 3 (Figure 5) demonstrated the most promising binding energy, as well as favorable interaction by hydrogen bonds with the amino acid Asn602 [39]. Kumar et al., studied the affinity of antipsychotic drugs towards NMDAR (PDB#1PBQ) by molecular docking to evaluate its potential use in the treatment of AD [40]. In the hydrophobic pocket of NMDAR 3D structure, the main residues are the Phe16, Phe92, Trp223, and Phe250 [40]. For the antipsychotic drug anisopirol (Figure 5), the compound of this group with the highest affinity mainly interacted with Phe92, Pro24, Thr126, Ser180, Trp223, and Phe246 [40]. Considering that this drug shared some interactions with the known NMDAR antagonist 5,7-dichlorokynurenic acid (DCKA), the co-crystallized ligand of this crystal structure, it might indicate that could also biologically interact with this target [40]. On the other hand, Singh et al. developed pharmacophore models based on the structure of ifenprodil, also a known NMDAR antagonist, performed virtual screening, studied the affinity of the hits by molecular docking and molecular dynamics simulations using the 3D structure of the NMDAR (PDB#5EWJ), as well as analyzed the hits ADMET properties [41]. The proposed study revealed that the molecules ZINC25726161 and ZINC95977857 (Figure 5) displayed a better affinity towards the target than the NMDAR antagonist drug ifenprodil, indicating that these virtual hits could have pharmacological interest [41].

### 2.3. Secretases

The proposed amyloid hypothesis states that the accumulation of the oligomeric form of Aβ peptide is the main cause of neuronal death and neurotoxicity in AD [42,43]. The processing of the amyloid protein precursor (APP) is regulated by several proteins, including secretases, specifically α-, β-, and γ-secretases, by two main pathways: the non-amyloidogenic and the amyloidogenic [44]. The α-secretase is mostly involved in the non-amyloidogenic pathway, cleaving APP into a APPα peptide and a smaller membrane-bound fragment, preventing the formation of Aβ [45]. Despite the fact that the exact role of α-secretases is still not fully understood, an increased production of APPα peptide could be a promising therapeutic strategy, due to its neuroprotective properties; however, no studies were found employing in silico approaches in the last five years [45]. On the other hand, in the amyloidogenic pathway, the APP is first cleaved by β-secretase, releasing two fragments that are processed by γ-secretase into Aβ_40/42_ and that have a higher tendency to aggregate and cause degeneration of neuronal cells [44]. Given the involvement of these enzymes, especially β-secretase, in APP processing, compounds that can interfere in this pathway can be of interest in AD treatment.

#### 2.3.1. β-Secretase

**Figure 6:**
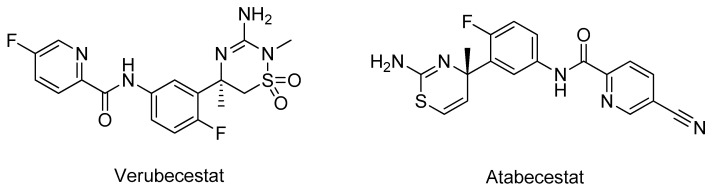
Structure of the BACE-1 inhibitors in clinical trials.

The β-site APP cleaving enzyme-1 (BACE-1, E.C. 3.4.23.46) is a β-secretase ubiquitously expressed in the brain that is involved in the amyloidogenic pathway [46]. The inhibition of BACE-1 is increasingly being viewed as a promising therapeutic strategy for AD drug development. Structurally, BACE-1 has a relatively large substrate-binding domain with affinity for several substrates, making the development of small molecules inhibitors to occupy such a large size more challenging [47]. Furthermore, the BACE-1 proteolysis is an intracellular process; thus, the inhibitors need to cross the cellular membranes to inhibit Aβ peptide production [48]. Numerous promising compounds advanced for clinical trials in the last years, but all of them failed, for example verubecestat (Phase III) and atabecestat (Phase II/III), displayed in Figure 6, because no clinical benefit was observed in patients [49]. 

**Figure 7:**
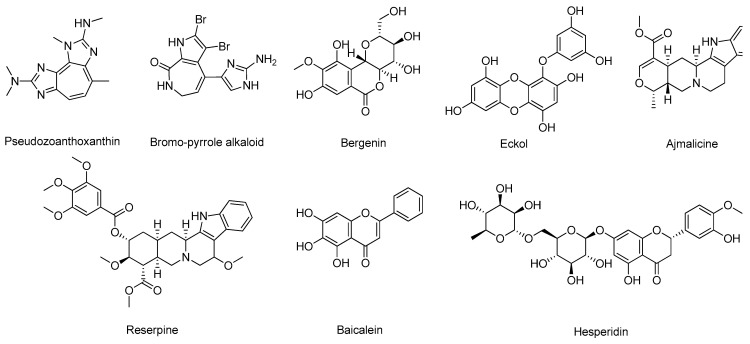
Structures of the studied BACE-1 inhibitors.

Gueto et al. employed a semiempirical method combined with molecular dynamics simulations to study the structural differences in the catalytic site of the BACE-1 (PDB#1FKN) when inhibitors with different potencies bind [50]. This study suggested that the residues Asp93, Asp289, Thr292, Thr293, Asn294, and Arg296 are key interactions for the ligand, accounting for almost half of the total protein-ligand interactions, indicating that if a compound interacts with these amino acids, it has a higher probability to inhibit BACE-1 [50]. Vitale’s group carried out a molecular docking analysis to evaluate the affinity of marine natural products with a 2-aminoimidazole aromatic group moiety towards the BACE-1 catalytic site (PDB#2QZL) in both “open” and “closed” states [51]. The studied compounds, pseudozoanthoxanthin and bromo-pyrrole alkaloid (Figure 7), were predicted to have affinity for both states of BACE-1 structure interacting with the main amino acids of the catalytic site. Specifically, most of the formed bonds were originated with the 2-aminoimidazole scaffold [51]. A simpler strategy was carried out by Barai and co-workers, which studied by molecular docking the interactions formed between the natural product bergenin (Figure 7) and BACE-1 (PDB#1FKN) and identified the main amino acids involved in the active site: Asp32, Gly34, Pro70, Tyr71, Thr72, Gln73, Asp228, Gly230, Thr231, and Arg235. Interestingly, they demonstrated that bergenin interacted with most of these amino acids, indicating that it can be a potential new inhibitor [52]. A similar methodology was performed for other natural compounds. For instance, Lee et al. analyzed several phlorotannins that interacted also with some of the residues above referred, especially eckol (Figure 7) [53]. Kashyap et al. observed that two plant natural compounds, reserpine and ajmalicine (Figure 7), also interacted with some of the most relevant amino acids, specifically Asp32, Thr72, and Asp228, of the “closed” state 3D structure (PDB#4D8C) [54]. Han’s team evaluated the affinity of baicalein (Figure 7) towards BACE-1 (PDB#2WJO), and observed an important interaction with the residue Ser35 of the catalytic site [55], while Jun et al. studied several citrus flavanones (Figure 7) for the same target, of which hesperidin (Figure 7) displayed the best results [56].

**Figure 8:**
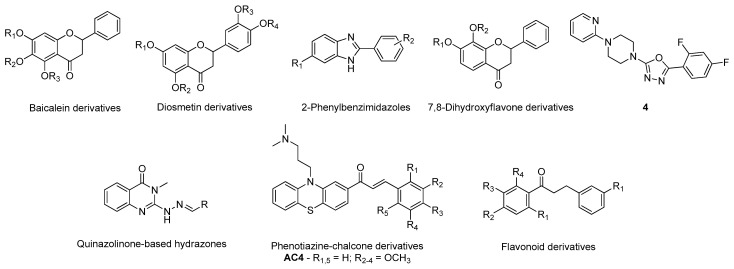
Structures of the studied BACE-1 inhibitors.

Similar to what occurs for synthetic compounds, flavone derivatives (PDB#6EQM), specifically baicalein and diosmetin derivatives [57], 2-phenylbenzimidazoles (PDB#1FKN) [58], 7,8-dihydroxyflavone derivatives (PDB#2ZHS) [59], molecular hybrids of 2-pyridylpiperazine and 5-phenyl-1,3,4-oxadiazoles (PDB#2ZJM), especially compound 4 [60], and quinazolinone-based hydrazones (PDB#4B70) [61], also have demonstrated very promising in silico results that can result in novel drug candidates for BACE-1 inhibition, as shown in (Figure 8). Tran et al. [62] built a 2D-QSAR model (R^2^ of 0.83) based on the structure of BACE-1 inhibitors found in the literature to identify novel potential inhibitors in a library of chalcone derivatives. The highest-ranking hits were submitted to molecular docking against BACE-1 (PDB#5HU1) to select the most promising candidates [62]. In particular, compound AC4 (Figure 8), a phenotiazine-chalcone derivative, displayed the highest affinity towards the target [62]. A distinct procedure was carried out by Subramanian and co-workers, using LBDD to construct a predictive model for small BACE-1 inhibitor molecules [63]. Gathering all the small inhibitors used to co-crystallize all the existing crystal structures of BACE-1, the team built 1-/2- and 3D-field descriptors and applied machine learning techniques to classify potential BACE-1 inhibitors [63]. On the other hand, Thai et al. validated a 2D-QSAR model built out of pharmacophoric 3D-models based on the structure of clinically used drugs and compounds in clinical trials to evaluate novel curcumin and flavonoids derivatives (Figure 8) based on their potential to inhibit BACE-1 [64]. Salvador’s research team [65] developed a model to also identify novel potential BACE1 inhibitors, but in this case combining LBDD with SBDD methodologies. Here, the research team employed pharmacophores and molecular docking-based virtual screening to discover new leads that were further submitted to docking analysis (PDB#2QP8) and filtered based on their predicted ability to cross the blood-brain barrier (BBB) to be selected for in vitro testing [65].

#### 2.3.2. γ-Secretase

**Figure 9:**
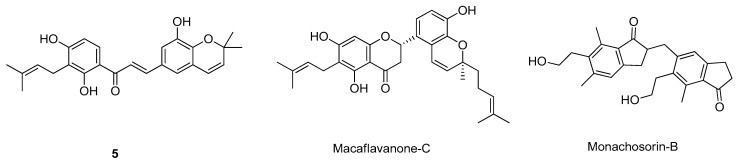
Structures of the studied γ-secretase inhibitors.

γ-Secretase is a multi-subunit protease enzyme complex involved in APP cleavage in the amyloidogenic pathway into Aβ peptide [66]. This protein complex is composed of presenilins, 1- (PSEN-1) and 2- (PSEN-2), presenilin enhancer 2, anterior pharynx-defective phenotype-1, and nicastrin [45]. Of these, PSEN-1 and -2 form the catalytic site and have a key role in APP processing, while the other proteins are mostly stabilizers of the complex [45,66]. Hence, γ-secretase inhibitors to decrease the formation of Aβ can be interesting drug candidates, especially compounds that can interact with PSEN-1 and -2 [45]. The existing γ-secretase inhibitors are associated with harmful side effects, such as hematological and gastrointestinal toxicity, skin reactions, and changes in hair color, caused by the inhibition of the γ-secretase on the metabolization of non-amyloid substrates [45]. Currently, the inexistence of available crystal/NMR structures of the γ-secretase in the PDB makes the in silico studies more challenging for this target [13]. However, Gupta et al. built a 3D structure by comparative homology modeling and studied the interactions formed with more than 4000 phytochemicals by docking and molecular dynamics simulations, identifying three potential drug candidates, compound 5, macaflavanone-C, and monachosorin-B (Figure 9) [67]. Hitzenberger et al. generated a structural model of γ-secretase bound to a L- 685,458 transition state inhibitor and predicted the possible locations and nature of the amino acids in the proposed binding pockets of the protein by docking and molecular dynamics simulations [68]. Both models might lead to the design of novel γ-secretase inhibitors drug candidates.

### 2.4. Sirtuins

**Figure 10:**
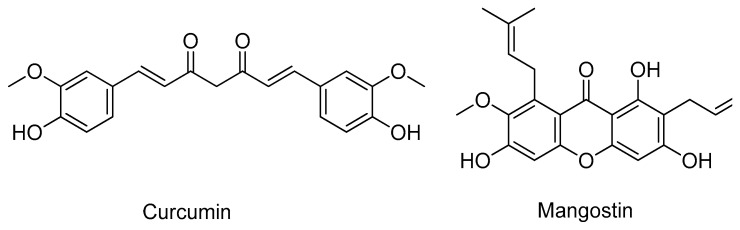
Structures of the studied SIRT inhibitors.

Sirtuins (SIRT) are a family of signaling and regulating proteins involved in many essential biological pathways that recently have been associated with the pathogenesis of several age-related diseases, including AD [69]. So far, seven SIRT have been identified and characterized in humans, but SIRT2 is considered the most important because it follows a tendency to accumulate in aging brains that could be linked to neurodegeneration [70]. As Suzuki et al. demonstrated in vitro and in vivo, the inhibition of SIRT2 showed a neuroprotective influence, making SIRT2 inhibitors compounds of potential therapeutic interest for AD [71]. Despite the existence of several human SIRT2 crystal structures, none has substrates/inhibitors-bound, making it more challenging to perform CADD studies with this target [72]. Despite these hurdles, Bren et al. performed an inverse molecular docking in order to screen the affinity of curcumin (Figure 10) against all the available human protein structures on the PDB [73]. The 3D structures of SIRT1 and -2 were predicted as potential targets for this compound, indicating a potential novel SIRT inhibitor [73]. Yeong and co-workers also studied the affinity of natural products, specifically compounds extracted from the plant *Garcinia mangostana* with known in vitro and in vivo SIRT2 inhibitory activity, through molecular docking against SIRT2 (PDB#3ZGV) [74]. Of these natural products, mangostin (Figure 10) formed hydrogen bonds with the residues of the active site Gly86, Gly261, Asn286, and hydrophobic interactions with the residues Asp95 and Glu288 [74].

### 2.5. Caspases

**Figure 11:**
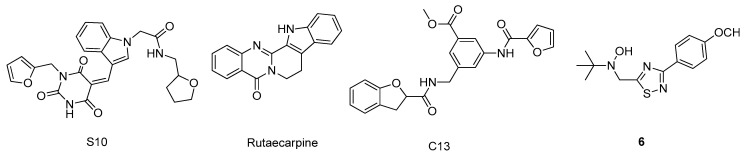
Structures of the studied caspases inhibitors.

Caspases are cysteine proteases that have essential roles in programmed cell death and axonal degeneration and exist in two main types: initiator and executioner [75]. The initiators, caspase-8, -9, and -10, are activated by autocatalytic cleavage, and then cleave and activate the executioners, caspase-3, -6, and -7, leading to cellular apoptosis [76]. Recently, multiple studies reported that executioner caspases, mostly caspase-6, of AD patients triggered uncontrolled apoptosis due to an excess activity, leading to a higher accumulation of the Aβ peptide [76]. Thus, the development of selective executioner caspase inhibitors could help regulating this dysfunction, and therefore, are also being considered potential drug candidates for AD [77]. However, several studies also explored the potential of new drugs candidates that target the initiator caspases for the treatment of AD [77]. In this context, LeBlanc et al. [78] performed a docking-based virtual screening of more than 57,000 small organic compounds against the 3D structure of caspase-6 (PDB#2WDP), specifically targeting the predicted allosteric binding pocket that contains the residues Ala34, Glu35, and Ala109. The predicted hits were submitted to another molecular docking analysis to evaluate their binding poses as well as verifying the interactions with the previously mentioned amino acids. Specifically, compound S10 (Figure 11) showed the highest binding energy and, therefore, was considered the most promising drug candidate [78]. Ahmad and co-workers [79] used a very similar strategy, but, in this case, to study the affinity of natural compounds towards caspase-8 (PDB# 1QTN). With a virtual screening of over 200 compounds, rutaecarpine (Figure 11) was predicted as the best hit, interacting with the amino acids of the catalytic site, Thr337, Lys353, Val354, Phe355, and Phe356. Kumi et al. [80] studied the binding mode and the atomic interactions formed with the allosteric binding site of caspase-6 (PDB#6DEV) of a specific inhibitor, compound C13 (Figure 11) by molecular dynamics, similar to the methodology that Cancela et al. [81] used to study the affinity of several nitrones against caspase-3 (PDB#1RHM). In this case, the nitrone that demonstrated the most promising results was compound 6 (Figure 11), binding in the substrate binding cleft and interacting with Arg207 by hydrogen bonds.

### 2.6. Glycogen Synthase Kinase-3

**Figure 12:**
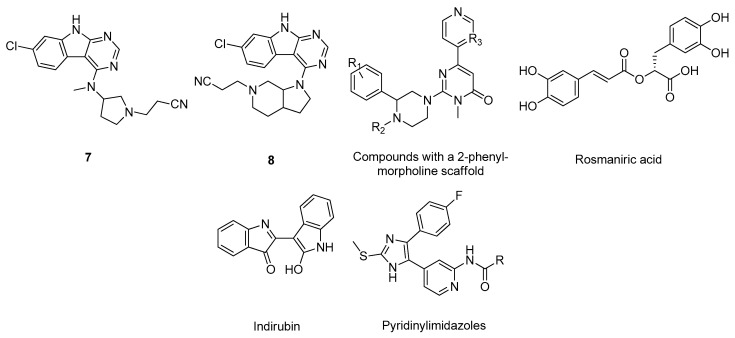
Structure of the studied GSK-3 inhibitors.

The glycogen synthase kinase 3 (GSK-3, E.C. 2.7.11.1) is a serine/threonine kinase regulator of several essential cellular processes that is involved in neuronal development, synaptogenesis and cell survival [82]. GSK-3 substrates include a wide variety of metabolic proteins, including APP, structural proteins, such as tau, and also transcription factors (e.g., Nox) [83]. Several studies reported that in the brain of AD patients, GSK-3 is over-expressed, contributing to a hyperphosphorylation of tau protein and to AD progression [84]. Hence, its inhibition is becoming a very promising therapeutic strategy to treat AD [84]. For example, Andreev et al. evaluated the affinity of several synthetic derivatives of the pan-Janus kinase inhibitor tofacitinib that inhibited in vitro GSK-3 through molecular docking and molecular dynamic simulations (PDB#3LXK) [85]. The most potent derivatives in vitro, compounds 7 and 8 (Figure 12), displayed very similar interactions with the GSK-3 active site, similar to the observed for compound tofacitinib, specifically with the residues Asp133, Val135, and Gln185 [85]. Identical methodologies were carried out by other researchers, in some cases employing molecular dynamics simulations, to assess the binding poses and affinities towards the GSK-3 active site for several compounds: derivatives with a 2-phenylmorpholine scaffold (PDB#3F88) [86], rosmarinic acid (PDB#1PYX) [87], indirubin (PDB#1I09) [88], and pyridinylimidazoles (PDB#4PTC) [89] as shown in Figure 12.

**Figure 13:**
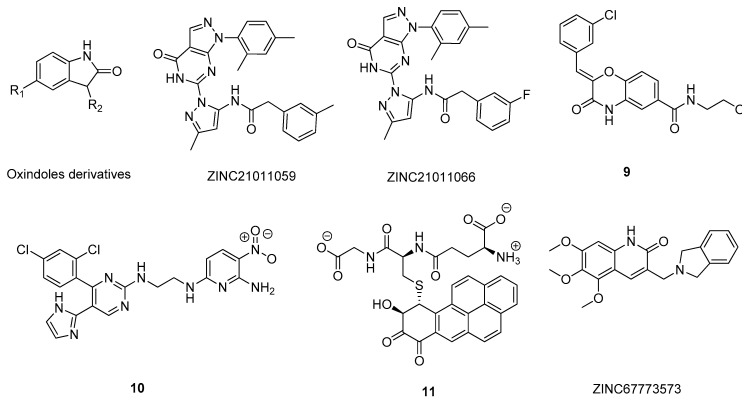
Structure of the studied GSK-3 inhibitors.

On the other hand, Lozinskaya and co-workers studied the affinity of oxindole derivatives by molecular docking but targeting the ATP-binding site of GSK-3 (PDB#4J1R) to identify potential new allosteric GSK-3 inhibitors [90] (Figure 13). Shukla et al. [91] performed a four-step virtual screening from sets of 5, 36, and 709 compounds to predict the most potent compounds for further docking against GSK-3 (PDB#1J1B) and ADMET properties analysis. After this, the top 29 compounds were submitted to molecular dynamics simulations, where compounds ZINC21011059 and ZINC21011066 (Figure 13) were predicted as being the most energetically favorable and with the highest potential to inhibit GSK-3 in vivo [91]. Joshi et al. [92] also employed docking-based virtual screening studies to identify new GSK-3 inhibitors from a library of over 50,000 small molecules, selecting several compounds with very promising in vitro results, for example compound 9 (Figure 13). He et al. [93] developed a 3D-QSAR (R^2^ > 0.95) model based on the structure of seventy-nine (5-imidazol-2-yl-4-phenylpyrimidin-2-yl)[2-(2-pyridylamino)ethyl] amine derivatives with GSK-3 inhibitory activity to discover potential new drug candidates. The hits were submitted to docking and molecular dynamics simulations to identify the specific interactions formed with GSK-3 (PDB#1J1B), as well as predicting the ADMET properties to select the top 10 drug candidates, of which compound 10 was the best ranked [93] (Figure 13). Natarajan’s group [94] used a slightly different approach, particularly a SBDD methodology where 20 crystal structures were used to generate pharmacophore models and screened in databases of small molecules to identify new potential inhibitors with positive ADMET properties. The resulting 2423 potential hits were analyzed by a combination of dockings, such as rigid receptor docking, quantum polarized ligand docking and induced fit docking, and with molecular dynamic simulations that showed that most of the hits interacted with the main residues of the active site of the GSK-3, especially compound 11 (Figure 13) [94]. Kerdawy and co-workers [95] carried out almost the same strategy, but with a smaller library set, 1250 compounds, filtering the structures that were predicted as being BBB permeable and had desirable pharmacokinetic properties. The top 25 compounds displayed positive binding poses and interacted with key amino acids (PDB#1Q4L) of the GSK-3 catalytic site, Asp133 and Val135, with good affinities, especially the quinoline-2-one derivative compound ZINC67773573 (Figure 13) that was the most promising candidate for novel GSK-3 inhibitors [95].

## 3. Parkinson’s Disease

PD is characterized by a progressive impairment of the voluntary motor control, as a consequence of the accumulation of α-synuclein-containing Lewy bodies in the *substantia nigra pars compacta* of the brain and the loss of dopaminergic neurons, leading to a decline in the levels of dopamine [96]. The primary clinical manifestations of PD include muscle rigidity, tremors, bradykinesia, and impaired postural reflexes that tend to worsen with the disease progression [97]. The current drug therapy for PD is only for symptomatic relief and is mainly focused on restoring dopaminergic function in the brain [97]. To date, the most effective drug for symptomatic treatment is levodopa (*L*-DOPA) combined with catechol-*O*-methyltransferase (COMT) and aromatic amino acid decarboxylase inhibitors. However long-term *L*-DOPA administration is usually associated with dyskinesia and motor fluctuations [97]. In most cases, a combination of several single-target drugs is used to enhance their pharmacological benefits, for example dopamine agonists, monoamine-oxidase (MAO) type-B inhibitors, amantadine, among other drugs [97]. Specifically, amantadine, a NMDAR antagonist, is used in PD treatment to increase the amounts of dopamine released in the brain, being used in an early stage of the disease to improve slow movements and muscle rigidity [98]. Also, clinical trials showed a short-term anti-dyskinetic effect of amantadine in patients with advanced PD [99]. On the other hand, primidone, an anti-convulsant drug, can be useful to treat essential and resting tremors in PD [100], and clozapine, an antipsychotic drug, can be used to address the potential psychosis of PD individuals and is also useful due to its anticholinergic properties [101,102]. Despite the intense research and investment in PD drug development, only a couple of adjunct new treatments were approved in the last decades, probably due to the multifactorial PD pathophysiology [6]. In the last years, several α-synuclein antibodies reached clinical trials phases, some for the disease diagnosis and others as potential immunotherapeutic agents for the treatment, but all of them were suspended [103]. Considering the diversity of the α-synuclein proteoforms and their distinct clinical/pathological relevance, additional studies are required to further develop novel antibodies with increased selectivity for these pathogenic and misfolded forms of this protein to enhance the clinical efficacy of this promising strategy [104].

Thus, there is an urgent demand for the development of novel drug candidates that address the limitations of currently used dopamine replacement treatments [6]. Therefore, in this subtopic, a detailed characterization of the advances accomplished in PD drug development in the last 5 years for several targets involving in silico approaches will be conducted. In this context, it is important to mention that among several relevant targets, in the past years, no significant progresses were achieved in the computational design of novel drug candidates of the anticholinergics class as well as amantadine analogues and consequently, they will not be described in this review.

### 3.1. Monoamine Oxidase Type B Inhibitors

**Figure 14:**
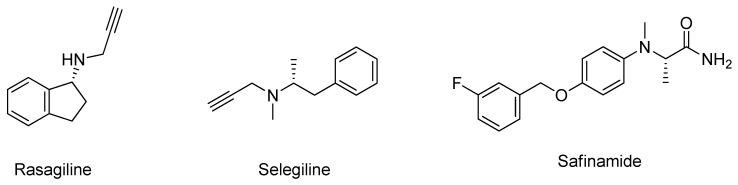
Structures of the clinically approved MAO-B inhibitors.

The MAO flavin enzyme catalyzes an oxidative deamination of biogenic and xenobiotic amines, including in the neurotransmitters dopamine, noradrenaline and serotonin [105]. MAO can be expressed in two distinct isoforms in humans: MAO-A and MAO-B, each presenting different substrate affinities and tissue distribution; however, MAO-B is considered the main pharmacological target in PD mainly due to its involvement in the dopamine deamination in the brain [106]. Also, with ageing, increased expression levels of MAO-B are observed in the brain, which leads to a higher dopamine metabolization and a higher production of hydrogen peroxide, which promotes the apoptosis of dopaminergic neuronal cells [106]. Considering the active role of MAO-B in dopamine metabolism, selective inhibitors of this enzyme are of interest in PD treatment [106]. Currently, three molecules are clinically approved, rasagiline, selegiline, and safinamide (Figure 14), to be used as adjuncts to *L*-DOPA therapy [106]. Safinamide reversibly inhibits MAO-B and decreases the abnormal glutamate release by modulating the channels of potassium and sodium ions. An ideal candidate for combination with safinamide is opicapone. This peripheral COMT inhibitor supports the continuous administration of *L*-DOPA in the brain and, therefore, the concept of continuous dopaminergic stimulation. Both compounds, with their application once a day and good tolerability, can complement each other by reducing the required oral levodopa intake and “OFF” times [107]. Despite the symptomatic relief that both offer, PD drug development is still focused on discovering novel and more effective MAO-B inhibitors [6]. Structurally, the MAO-B catalytic site contains the amino acids Leu171, Ile199, and Tyr326, which are the “gatekeeper” residues that maintain the functional conformation of the active site, whereas the residues Tyr60, Gln206, Tyr398, and Tyr435 are involved in substrate-binding [108].

**Figure 15:**
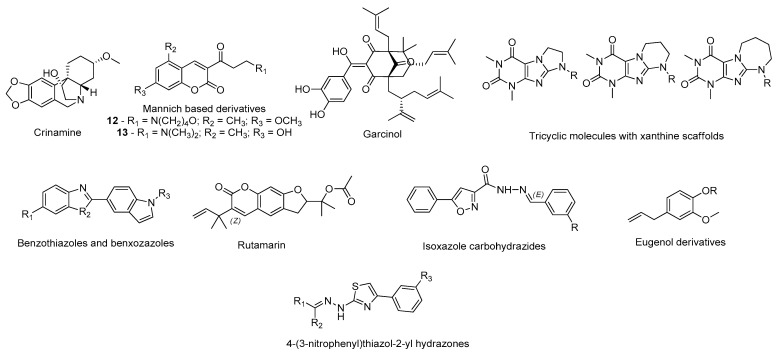
Structures of the studied MAO-B inhibitors.

For instance, Naidoo et al. studied the affinity of several biologically active constituents of the plant *Crossyne guttata*, with in vitro inhibitory MAO-B activity, against the catalytic site of MAO-B (PDB#2BYB) through molecular docking [109]. Of these, the alkaloid crinamine (Figure 15), with favorable ADMET properties, displayed the highest binding energy and also interacted with the residues Leu171, Ile199, Tyr326, and Tyr 435. Therefore, this alkaloid can be a potential drug candidate in this context [109]. Tao and co-workers also performed a molecular docking study to analyze the atomic interactions formed between synthetic coumarin Mannich base derivatives and the MAO-B active site (PDB#4A79) [110]. Interestingly, the compounds with a higher in vitro inhibitory activity, 12 and 13 (Figure 15) were also the molecules with the highest affinity towards the catalytic site of MAO-B, interacting with the main amino acids of the active site (Tyr60, Leu171, Ile198, Tyr 398, and Tyr435) [110]. Similar strategies were carried out by other researchers to study the drug potential of garcinol (PDB#3PO7) [111], tricyclic molecules with xanthine scaffolds (PDB#2V5Z) [112], benzothiazoles and benzoxazoles (PDB#2V5Z) [113], rutamarin (PDB#2V60) [114], isoxazole carbohydrazides (PDB#2V60) [115], eugenol derivatives [116], and 4-(3-nitrophenyl)thiazol-2-yl hydrazone derivatives (PDB#6FW0) [117] in PD, as summarized in Figure 15.

**Figure 16:**
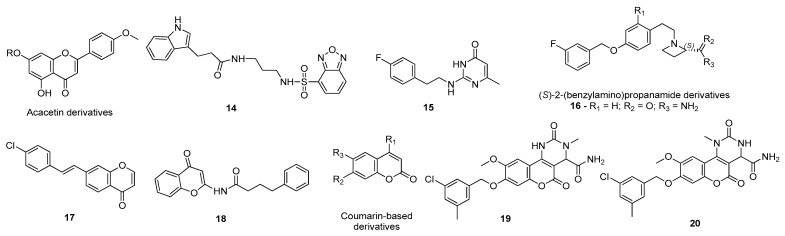
Structures of the studied MAO-B inhibitors.

Moreover, the Chaurasiya research team also studied the affinity of several acacetin derivatives (Figure 16) by molecular docking and combined this analysis with molecular dynamics simulations to evaluate the compounds’ binding modes and selectivity towards both MAO-A (PDB ID: 2Z5X) and MAO-B (PDB#4A79) isoforms [118]. According to the in silico results, the referred compounds displayed higher affinity against MAO-B than MAO-A and the binding orientations were very identical to those performed by native ligands of the 3D structures [118]. Of these, acacetin 7-methyl ether (Figure 16) was the compound that displayed the most promising results, interacting with Ile199, Ile316, and Tyr326, which are critical residues for MAO-B selectivity [118]. Furthermore, the molecular dynamics simulations suggested highly stable binding poses in MAO-B binding pocket [118]. Is et al. virtually screened a library of 256,750 molecules to discover novel MAO-B inhibitors using SBDD and LBDD methodologies [119]. The compounds were ranked based on their docked binding affinities for MAO-B (PDB#1S3B) and were further filtered by a binary QSAR model, which selected the compounds with the most favorable ADMET properties [119]. After this study, two compounds, compounds 14 and 15 (Figure 16), advanced for further analysis by molecular dynamics simulations to investigate their structural and dynamic properties [119]. Of these, ligand **15** was predicted as being the compound with the highest binding energy, most adequate ADMET properties, and a higher potential to inhibit MAO-B [119]. Jin and co-workers created a computational protocol to discover new selective inhibitors of this enzyme through fragment-based drug design based on the binding mode and selectivity of safinamide for the MAO-B active site (PDB#2V5Z) [120]. Therefore, a fragment-based virtual screening was performed and the hits with the most favorable ΔG values advanced for further in vitro studies, confirming that predicted (*S*)-2-(benzylamino)propanamide derivatives (Figure 16) displayed a relevant MAO-B inhibitory potential [120]. Furthermore, compound 16 containing a chiral azacyclic amide moiety was considered the molecule with the highest potential to inhibit MAO-B, being also demonstrated in vitro [120]. Cruz-Monteagudo et al. conducted a cheminformatics analysis to evaluate the potential of chromone derivatives and analogues as MAO-B inhibitors [121]. Based on the information of several SAR studies, two relevant chromone systems were discovered, compounds 17 and 18 (Figure 16) [121]. Both compounds displayed high affinity towards the enzyme catalytic site (PDB#2V61), as demonstrated by molecular docking [121]. The Mladenović research team also developed an extensive in silico methodology to develop novel MAO-B inhibitors using structure-based 3D-QSAR models, built using the structures of known MAO-B inhibitors deposited in the PDB, and as training sets the structures of compounds with a wide-ranging molecular diversity (aminoindans, aromatic amines, aliphatic amines, aryloxybenzenes, coumarins, thiazolidine-2,4-diones, indoline-2,3-diones, 1,4-diphenyl-2-butenes, terpenes, and imidazolines) [122]. From a set of 128 novel coumarin-based inhibitors, 18 compounds were selected and tested by molecular docking, of which the compounds 19 and 20 (Figure 16) were considered the most promising drug candidates [122].

### 3.2. Dopamine Agonists

**Figure 17:**
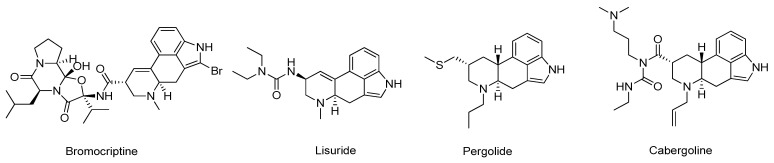
Structures of the ergoline class clinically approved dopamine agonists.

Dopamine receptors are members of the G-protein-coupled receptors that can be subdivided into two main groups based on their pharmacological behavior: D_1_ (D_1_ and D_5_) and D_2_ (D_2_, D_3_ and D_4_) type receptors [123]. In particular, dopamine agonists (DA) are compounds that can directly activate dopamine receptors, relieving PD symptoms related to the low levels of the neurotransmitter [123]. The current clinically approved DA mainly target the D_2_-type dopamine receptors and can be divided into two main subclasses, ergoline and non-ergoline DA (Figure 17) [124]. The drugs of the ergoline class, which include bromocriptine, lisuride, pergolide, and cabergoline, are rarely recommended due to their harmful side effects.

**Figure 18:**
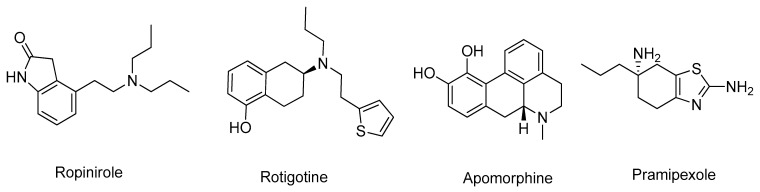
Structures of the ergoline class clinically approved DA.

Nonetheless, the more recent non-ergoline class, which includes ropinirole, rotigotine, apomorphine, and pramipexole (Figure 18), are the DA most commonly used for symptomatic relief in PD [124]. Currently, DA are only a therapeutic option for younger patients in an attempt to delay the onset of *L*-DOPA therapy and are not recommended for individuals over 60 years old due to their adverse side effects [125]. Thus, researchers are focused on the design of more effective and less toxic DA to improve PD treatment [125].

**Figure 19:**
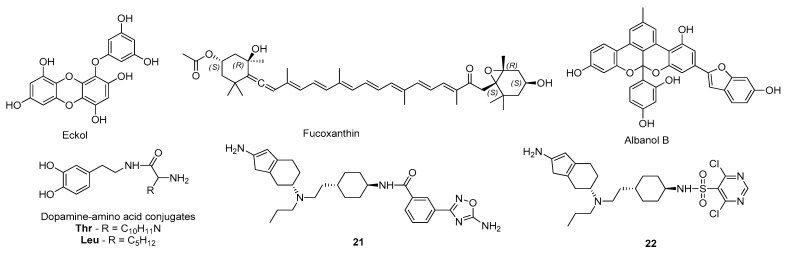
Structures of the studied DA.

Paudel and co-workers investigated the effects of eckol (Figure 19), a marine natural algae-product, in dopamine receptors as a potential PD drug candidate [126]. For this, docking and molecular dynamics simulations specifically targeting the active site of the dopamine receptors D_3_ (PDB#3PBL) and D_4_ (PDB#5WIU) were performed [126]. For the dopamine receptor D_3_, eckol formed interactions similar to the determined for rotigotine, particularly a hydrophobic interaction with the residue Val107 and a hydrogen bond with Asp110, but a lower binding energy was estimated [126]. Regarding the dopamine receptor D_4_, in comparison with nemonapride, eckol also displayed lower binding energy and formed similar interactions with the residues Asp115, Cys119, Val193, and Phe410 [126]. Besides, the molecular dynamics simulations for both macromolecular targets showed stable binding poses, mainly due to a π–π interaction with the amino acid Phe346 [126]. Considering these results and the ADMET properties of this compound, which were also predicted as being generally favorable, eckol might be considered a potential DA drug candidate, acting in dopamine receptors D_3_ and D_4_ [126]. The same research group used a similar strategy to study the affinity of fucoxanthin and fucosterol, two other marine algae-products, against the dopamine receptors D_3_ (PDB#3PBL) and D_4_ (PDB#5WIU) [127]. Overall, based on in silico and in vitro studies, it was demonstrated that fucoxanthin (Figure 19) has a potent D_3_/D_4_ agonist activity that might be effective in the management of PD symptoms [127]. Recently, the same group evaluated Diels–Alder type adducts from *Morus alba* root bark, with known mild to moderate MAO inhibitory activity, as potential DA [128]. For this, molecular docking was performed against the dopamine receptors D_2_ (PDB#6CM4), D_3_ (PDB#3PBL), D_4_ (PDB#5WIV), and D_1_ receptor, which was built by homology modeling from the β_2_-adrenergic receptor [128]. Generally, the results indicated that all the tested compounds have potential as DAs, especially albanol B (Figure 19), which showed the highest affinity for all dopamine receptors studied [128]. Furthermore, Tutone et al. built a homologue structure of the dopamine receptor D_1_ to further evaluate by molecular docking the affinity of dopamine-amino acid conjugates [129]. Moreover, molecular dynamics simulations were used to refine the model and also to predict the binding pocket of the catalytic site [129]. Of the tested structural variations, dopamine-Thr and dopamine-Leu (Figure 19) were considered the conjugates with the highest affinity, mostly due to interactions with the residues Asp103 and Asp314. Thus, they were considered the compounds with the highest potential to use as agonists of the dopamine receptor D_1_ in PD therapy [129]. Duan et al. used a comparative molecular field analysis (CoMFA) approach for 3D-QSAR (R^2^—0.982) and structure–selectivity relationship (3D-QSSR) (R^2^—0.876) models to discover new D_3_ receptor agonists [130]. An initial set of 40 D_3_ receptor agonists reported by Chen et al. [131] was used to build the 3D-QSAR/QSSR models based on the CoMFA method and the hits were submitted to molecular docking to evaluate its affinity towards the D_3_ receptor (PDB#3PBL) [130]. Based on the structure of the best ranked molecules, six hybrid molecules were designed, where the compounds 21 and 22 (Figure 19) displayed even more positive results, being considered potential compounds to be further studied in vitro and in vivo [130]. 

### 3.3. Adenosine Receptors Antagonists

**Figure 20:**
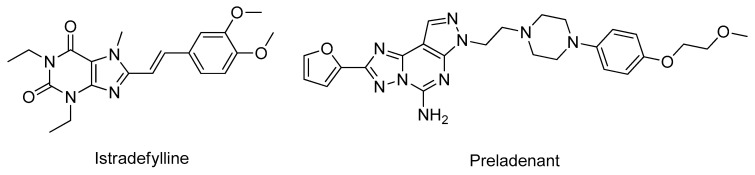
Structures of the clinically approved adenosine receptor A_2A_ antagonists.

Adenosine is an essential endogenous modulator of the CNS that interacts with G protein-coupled receptors, specifically with four adenosine receptors, the A_1_, A_2A_, A_2B_, and A_3_ types [132]. Specifically, the A_2A_ receptor is involved in motor control and is physically and functionally associated with the dopamine D_2_ receptor in the dopaminergic regions of the brain, being able to act as a brake for the D_2_ receptor signaling [133]. The blockade of A_2A_ receptors through antagonists increases D_2_-dependent signaling, enhancing the clinical effect of *L*-DOPA and reducing the dyskinesia associated with its long-term administration, as well as increasing the sensitivity of dopaminergic neurons and protecting against neurodegeneration [133]. Recently, multiple adenosine receptor A_2A_ antagonists have emerged as potential drug candidates for PD with some compounds entering clinical trials, including istradefylline and preladenant (Figure 20). However, none of these products originated a significant pharmacological benefit in PD patients and, therefore, the studies were suspended [133].

**Figure 21:**
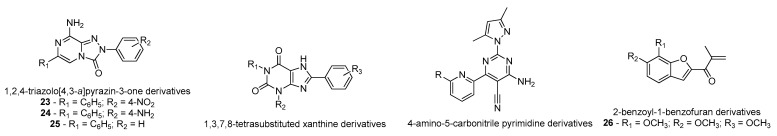
Structures of the studied adenosine receptor antagonists.

Falsini and co-workers investigated the potential of multiple compounds with a 1,2,4-triazolo[4,3*-a*]pyrazin-3-one scaffold as novel adenosine A_2A_ receptor antagonists by molecular docking (PDB#3EML and PDB#4EIY) [134]. The studies were performed with two crystal structures of A_2A_ receptors to better understand the compounds’ binding mode and to improve the reliability of the results [134]. The tested scaffold formed important hydrophobic interactions with Phe168 and hydrogen bonds with the residues Glu169 and Asn253, similar to those that the co-crystallized ligand ZM-241,385 present for the same targets [134]. Additionally, the affinities towards the adenosine A_1_ receptor (PDB#5UEN) and to a homologue model of the adenosine A_3_ receptor were also evaluated [134]. Interestingly, compounds 23 and 24 (Figure 21) showed the highest affinity for A_1_ receptor, and compound 25 displayed a higher affinity against all tested macromolecular targets, being considered a potential novel adenosine receptor antagonist with affinity for multiple receptors [134]. Załuski and co-workers designed new *N*9-benzyl-substituted imidazo-, pyrimido- and 1,3-diazepino[2,1-*f*]purinediones derivatives and evaluated their potential adenosine A_2A_ receptors antagonistic activity with in vitro and in silico approaches [112]. Thus, thirty-seven novel derivatives were studied by molecular docking to evaluate their affinity towards the target (PDB#3REY) [112]. The majority of these compounds interacted with the residues Phe168 and Phe263, important residues of the A_2A_ receptors binding pocket, indicating that all ligands fitted well in the catalytic site, which might be an explanation for the relevant in vitro antagonistic activities [112]. Similar strategies were carried out to investigate the potential of several other compounds as adenosine A_2A_ receptors antagonists such as: 1,3,7,8-tetrasubstituted xanthine derivatives (PDB#3EML) [135] and 4-amino-5-carbonitrile pyrimidine derivatives (PDB#3VGA) [136] (Figure 21), being predicted in silico to be selective and potent adenosine A_2A_ antagonists. On the other hand, Janse et al. employed in silico methodologies to predict the ADMET properties of methoxy substituted 2-benzoyl-1-benzofuran derivatives that showed very promising in vitro adenosine A_1_/A_2A_ receptors antagonist activity, in which compound 26 (Figure 21) displayed the best results [137].

### 3.4. Catechol-O-methyltransferase Inhibitors

**Figure 22:**
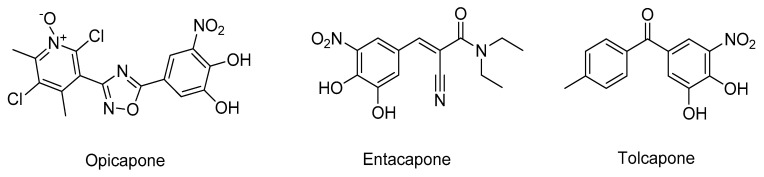
Structures of the clinically approved COMT inhibitors.

COMT is a ubiquitous enzyme responsible for the *O*-methylation of catechol substrates like dopamine, which is expressed in two molecular isoforms in humans: a soluble form (SCOMT) and a membrane-bound (MBCOMT), which is the predominant isoform in the brain [138]. Mainly due to the involvement of COMT in dopamine and *L*-DOPA metabolism, COMT has been increasingly associated with PD pathogenesis [138]. Particularly, near 90% of the *L*-DOPA administered is rapidly metabolized by COMT into 3-*O*-methyldopa before reaching the brain, therefore not exerting its pharmacological effect [138]. However, if COMT inhibitors are used as adjuncts to this therapy, a higher amount of *L*-DOPA reaches the brain and the degradation of dopamine is reduced, improving the clinical efficiency of this treatment [139]. Currently, only three COMT inhibitors are clinically used in PD treatment, opicapone, entacapone, and tolcapone (Figure 22) [139]. However, of these, only tolcapone can cross the BBB and inhibit brain COMT activity, but its clinical use is limited because of its high hepatoxicity [139].

**Figure 23:**
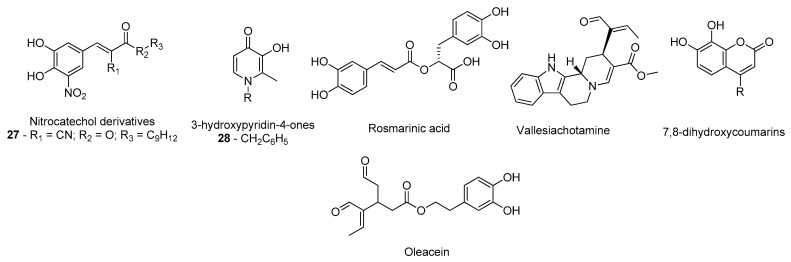
Structures of the studied COMT inhibitors.

Even with the improvements that these inhibitors brought to PD treatment, researchers and pharmaceutical companies are still focused on the development of more effective and safer COMT inhibitors to improve PD therapy [140]. Structurally, the COMT catalytic site is surrounded by the “gatekeeper” residues Trp43 and Pro174, which ensure the correct orientation of the substrate, the magnesium ion and the *S*-(*5*′-Adenosyl)-*L*-methionine (SAM) cofactors, as well as residues Trp143, Lys144, and Glu199, which are involved in substrate binding [141]. Silva et al. designed a series of novel nitrocatechol-based compounds (Figure 23), structurally identical to tolcapone, and evaluated their in silico affinity through molecular docking with COMT (PDB#2CL5) and also predicted their ADMET properties [142]. Of the synthesized compounds, nitrocatechol 27 exhibited appropriate ADMET properties and the highest binding energy of all the tested ligands, interacting with residues Lys144, Pro174, and Glu199 [142]. These predictions were later confirmed by in vitro studies, evidencing the role of the 1,2-dihydroxy-3-nitrobenzene moiety for high COMT inhibitory activity [142]. De Beer et al. also evaluated by means of molecular docking the affinity of several 3-hydroxypyridin-4-ones (Figure 23) that in vitro had displayed high inhibitory activity against COMT (PDB#3BWM), using the COMT inhibitor 3,5-dinitrocatechol as control [143]. Particularly, compound 28 displayed a binding pose very identical to 3,5-dinitrocatechol and shared some common interactions with the residues Trp38, Trp143 and Lys144 as well, indicating that with refined structure modifications a potential drug candidate could be designed [143]. Similar strategies were employed by other research groups to investigate the interactions formed in the COMT active site with other compounds like rosmarinic acid (PDB#3BWM) [144], vallesiachotamine (PDB#3BWM) [145], 7,8-dihydroxycoumarins (PDB#3BWY) [146], and oleacein (PDB#3BWM) (Figure 23), which combined docking with molecular dynamics simulations [147].

**Figure 24:**
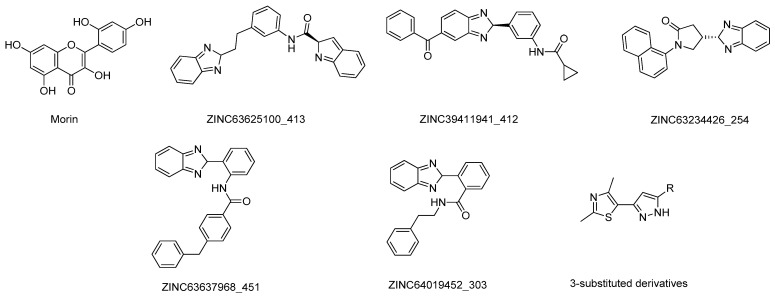
Structures of the studied COMT inhibitors.

Govindasamy et al. carried out a molecular screening to identify novel flavonoids as COMT inhibitors using a combination of docking, molecular dynamics, and QM/MM analysis with the 3D structure of COMT (PDB#3BYW) [148]. Interestingly, morin (Figure 24) was predicted from a set of 19 flavonoids as the compound with the highest docking and glide score, forming important interactions with the catalytic triad and the “gatekeeper” residues as well, indicating that it might be a potential drug candidate for PD treatment [148]. In addition, Patel et al. developed a pharmacophore model based on the structure of 23 known COMT inhibitors to perform virtual screenings of compounds of the ZICN database to identify novel potential leads [149]. Using filters such as being BBB permeable and positive ADMET properties, a final set of 36 possible leads were selected and their binding poses with the COMT active site (PDB#3A7E) were analyzed by docking and molecular dynamics simulations [149]. Of these, the compounds ZINC63625100_413, ZINC39411941_412, ZINC63234426_254, ZINC63637968_451, and ZINC64019452_303, displayed in Figure 24, have the highest score and can form important interactions with the active site, being considered potential COMT inhibitors [149]. Lerner et al. applied a fragment screening in silico approach to identify novel compounds that specifically targeted the SAM binding site of the COMT catalytic site (PDB#5K05) [150]. From an initial set of 6000 compounds, the structures that violated the Lipinski’s rules were filtered, 600 fragments were selected based on the affinity towards the target and submitted to further molecular docking studies [150]. This novel approach identified several moieties that were further studied in vitro and displayed high COMT inhibitory activity, which can be even further enhanced through structural optimization [150].

## 4. Conclusions

As the number of elderly increases, ND are becoming ubiquitous. Beginning with an overview of AD pharmacotherapy and existing blockbuster drugs, this review covers the potential of both natural and synthetic small molecules; the role of cholinesterases in the on-set and progression of AD and their inhibition; the role of BACE-1 in the production of β-amyloid proteins, one of the key reasons of the progression of AD; and other targets identified for AD drug discovery. Subsequently, we conducted the review addressing the several drugs that target PD, from the multiple adenosine receptor A2A antagonists that have emerged as potential drug candidates to MAO-B and COMT inhibitors, showing that some molecules might improve the clinical efficiency of PD treatment in a near future. In this reality, in silico techniques have proven to be essential and have become fundamental approaches in modern drug discovery, allowing for a better understanding of the diseases at a molecular level and to identify and validate these molecular targets. However, some of the compounds mentioned in this study have been withdrawn in different stages of drug development including in clinical trials because of receptor-protein non-specificity or ineffectiveness in human trials.

Nowadays, no effective treatments are available to prevent or cure AD nor PD, and the approved drugs only provide temporary and modest symptomatic improvements. However, in the upcoming years, a rise in the number of molecules under study for these pathologies is expected, and multi-target compounds for these ND will be increasingly studied. Particularly for ND, the drugs need to cross the BBB to exert their effects in the brain, which makes more challenging in terms of drugs accessibility. Thus, molecules designed and developed for common administration routes (e.g., oral) even after showing promising prediction results in in silico studies and favorable in vitro and in vivo data, often are not effective in situ. Thus, new routes of administration in humans must also be evaluated in clinical trials, such as inhalation, which will imply new algorithms for neuroimaging simulations to evaluate the potential of computationally predicted molecules and targets against AD and PD.
